# Evolution of Animal Parasitism in Nematodes of the Suborder Spirurina

**DOI:** 10.1002/ece3.73422

**Published:** 2026-04-07

**Authors:** Seiya Nagae, Koichi Hasegawa

**Affiliations:** ^1^ Department of Environmental Biology, College of Bioscience & Biotechnology Chubu University Kasugai Japan

**Keywords:** ancestral state reconstruction, cockroach, host–parasite coevolution, intermediate hosts, millipede, molecular phylogeny, oxyuridomorpha

## Abstract

The phylum Nematoda includes species adapted to nearly every environment on Earth, ranging from free‐living forms to parasites of plants and animals. Parasitism has evolved multiple times independently within the phylum, and the suborder Spirurina is particularly notable because all of its members are obligate animal parasites. In this study, we conducted a molecular phylogenetic analysis of Spirurina, integrating ancestral state reconstruction (ASR) to clarify higher‐level relationships and to trace the evolutionary origins of parasitism. Our analysis suggested that the parasitic origin of Spirurina, which arose from free‐living nematodes within Rhabditida that had adapted to terrestrial environments, is bifurcated. One is associated with early‐dividing lineages of aquatic invertebrates (Gnathostomatomorpha and Seuratoidea), while the other is associated with millipedes belonging to a large terrestrial clade that includes the suborders Oxyuridomorpha, Rhigonematomorpha, Ascaridomorpha, Camallanomorpha, and Spiruromorpha. Comparative examination of life cycles across infraorders indicates that parasitic strategies evolved from simple, single‐host infection cycles to complex life cycles requiring intermediate hosts. These complex cycles appear to have originated in freshwater environments, where copepods and other crustaceans served as ancestral intermediate hosts in aquatic lineages. Importantly, our findings identify millipedes as a pivotal ancestral host group that shaped the early terrestrial evolution of parasitism within Spirurina. Together, these findings provide a comprehensive framework for understanding the evolutionary pathways that gave rise to the remarkable diversity of parasitic strategies within Spirurina.

## Graphical abstract

This study reconstructs the evolutionary history of parasitism in Spirurina by integrating ancestral state reconstruction (ASR) with a comprehensive phylogenetic framework. Our analyses reveal clear patterns of hostswitching and major transitions in host associations, providing the first broad evolutionary perspective on parasitism within this group. These findings offer novel insights into how ecological interactions have shaped the diversification of parasitic nematodes.

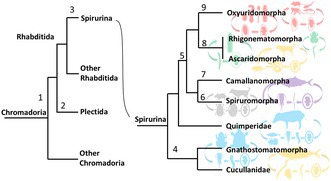

## 1 Introduction

**FIGURE 1:**
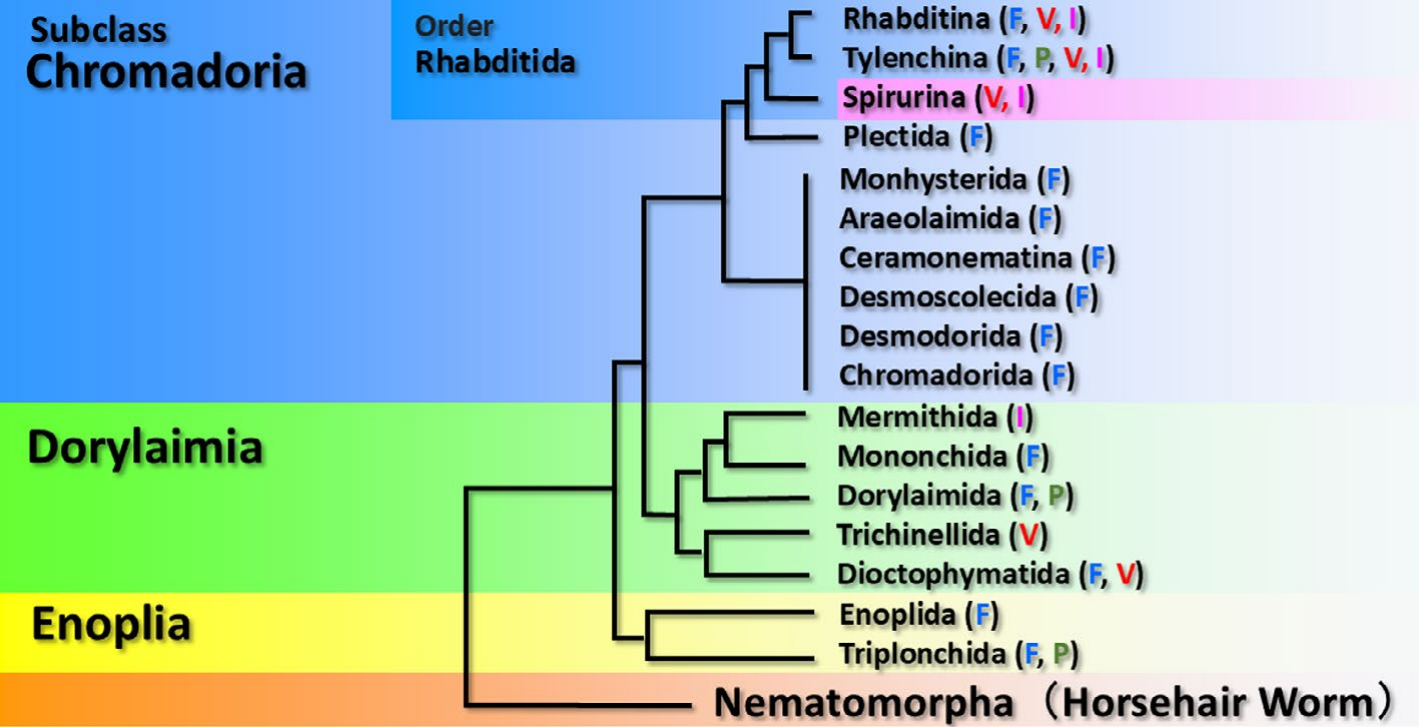
Phylogenetic tree of the phylum Nematoda, created based on the conceptual framework presented in Figure 1 of Blaxter and Koutsovoulos (2015) and Figure 3 of Ahmed et al. (2022). The tree follows classification names based on morphological taxonomy, and therefore some inconsistencies remain in the delineation of classes and orders. F, free living, microbivore (bacteria, fungi, and algae) and predator; I, invertebrate‐parasitic; P, plant‐parasitic; V, vertebrate‐parasitic.

Animals belonging to the phylum Nematoda have adapted to nearly every environment on Earth, occupying a wide range of ecological roles from free‐living forms to parasites of animals and plants (Blaxter and Koutsovoulos 2015). Although only around 30,000 species have been described to date, it is estimated that between 500,000 and 10 million species may exist, highlighting their remarkable diversity (Hodda 2022). Furthermore, with an estimated global biomass of approximately 0.3 gigatons and exceptionally high densities in terrestrial soils, nematodes are among the most numerically dominant animals on the planet (van den Hoogen et al. 2019). An early phylogenetic classification based on morphological characteristics divided the phylum Nematoda into two classes (Maggenti 1983, 1991), but molecular phylogenetic analyses have since reclassified it into three subclasses: Enoplia, Dorylaimia, and Chromadoria (Figure 1) (Blaxter and Koutsovoulos 2015; Ahmed et al. 2022). The subclass Chromadoria contains three particularly cohesive suborders: Rhabditina, which includes many soil‐dwelling nematodes; Tylenchina, which comprises numerous plant‐parasitic nematodes; and Spirurina, consisting exclusively of animal‐parasitic nematodes. These three suborders are grouped together within the order Rhabditida (Figure 1). Molecular phylogenetic analysis of Rhabditida, which encompasses these three suborders, suggests an evolutionary transition from a marine ancestor to freshwater, semi‐aquatic, and ultimately terrestrial habitats (Blaxter and Koutsovoulos 2015).

Animal parasitism has independently evolved in multiple lineages within the phylum Nematoda, yet the entire Spirurina group consists exclusively of animal‐parasitic species (Figure 1). Some members of Spirurina have complex life cycles involving multiple intermediate or paratenic hosts, whereas others have simple life cycles, directly infecting a definitive host without the involvement of intermediate hosts (Ando et al. 1992; Leung et al. 2020; Ozawa and Hasegawa 2018). In addition, several species cause diseases in humans, such as anisakiasis, ascariasis, gnathostomiasis, and guinea worm disease (Leung et al. 2020; Rahmati et al. 2020; Liu et al. 2020; Tayeh et al. 2017). Consequently, Spirurina represents an ideal model for studying the evolution of animal parasitism.

Of the six infraorders of Spirurina, Oxyuridomorpha is characterized by a simple life cycle and includes species that parasitize a diverse range of animal hosts (Adamson 1994). The group encompasses the superfamily Oxyuroidea (3 families, 4 subfamilies, 104 genes, 779 species), which parasitize vertebrates, and the superfamily Thelastomatoidea (5 families, 5 subfamilies, 90 genes, 479 species), which parasitize arthropods (Adamson 1994; Ozawa and Hasegawa 2018; Hodda 2022). A study examining intestinal parasitic nematodes in nine species (11 isolates) of millipedes from the family Xystodesmidae in Japan reported the presence of two phylogenetically distinct groups of parasitic nematodes: the superfamilies Thelastomatoidea and Rhigonematoidea (Nagae et al. 2021). Although both superfamilies belong to the suborder Spirurina, Thelastomatoidea is a member of the infraorder Oxyuridomorpha, whereas Rhigonematoidea belongs to the infraorder Rhigonematomorpha, which exclusively comprises millipede‐parasitic nematodes. These two groups coexist without competing with each other (Nagae et al. 2021). Since both groups exhibit simple life cycles without involving intermediate hosts (Adamson 1994; Hunt 1996), these parasitic nematodes may represent the most primitive lineage within the suborder Spirurina. Additionally, the superfamily Cosmocercoidea, previously classified under the infraorder Ascaridomorpha (Alcantara et al. 2022; Chen, Zhang, Feng, et al. 2020; Chen, Zhang, and Li 2020), has been proposed to belong to the infraorder Rhigonematomorpha (Nagae et al. 2021). This study also suggests that the superfamily Cosmocercoidea may present a polyphyletic group (Nagae et al. 2021).

As described above, the suborder Spirurina comprises parasitic nematodes with highly diverse host specificities and infection cycles, yet their phylogenetic relationships remain incompletely understood. In particular, it is unclear how host associations shifted across major lineages and how simple, single‐host infection cycles gave rise to more complex life cycles involving intermediate hosts. By integrating molecular phylogenetic analysis with ancestral state reconstruction (ASR) and ecological information, we aim to clarify the evolutionary pathways that shaped the remarkable diversity of parasitic strategies within Spirurina, with special emphasis on the infraorder Oxyuridomorpha.

## 2 Materials and Method

### 2.1 Molecular Phylogenetic Analysis of the Suborder Spirurina

To analyze the phylogenetic relationships among groups within the suborder Spirurina, we collected sequence data for the D2/D3 segment of the 28S ribosomal RNA gene (D2/D3 28S rRNA) and the 18S ribosomal RNA gene (18S rRNA) of nematodes from the NCBI database. These sequences represent nematodes from 15 superfamilies across six infraorders within Spirurina. Additionally, we obtained gene sequences from free‐living nematodes of the superfamily Rhabditoidea— *Caenorhabditis elegans* , *C. briggsae* , and *Oscheius tipulae—which* are considered to be the direct ancestors of Spirurina and used here as the outgroup. The hosts and infection cycles of parasitic nematodes are summarized in Table S1 from information listed in the NCBI database and published papers.

In addition, we included sequences from five newly identified species of parasitic nematodes belonging to the superfamily Thelastomatoidea, all of which inhabit millipedes. Multiple nematode species from the superfamilies Thelastomatoidea and Rhigonematoidea are known to parasitize millipedes (Nagae et al. 2021). Following the method of Nagae et al. (2021), we identified the host millipede species and isolated nematodes for morphological classification, gene sequencing, and infrapopulation surveys (data not shown). Genomic DNA was extracted from single nematode individuals, and sequences of the D2/D3 28S rRNA and 18S rRNA genes were obtained from three individuals per sequence. The resulting D2/D3 sequences were subjected to Blast searches in the NCBI database, and taxonomic identification at the family or genus level was determined based on sequence similarity, morphological characteristics, and host information of the closest matches. All sequences obtained from conspecific individuals were 100% identical; therefore, representative sequences were deposited in the NCBI database and are summarized in Table S2 together with the corresponding host data.

BioEdit version 7.2.6 (Hall 1999) was used to perform ClustalW multiple alignment, with sequence alignments automatically trimmed using the default settings of trimAI (Capella‐Gutiérrez et al. 2009). After gap processing, the D2/D3 28S rRNA and 18S rRNA sequences were concatenated to construct a phylogenetic tree (aligned dataset is available as Supporting Information 1). The phylogenetic tree of the suborder Spirurina was generated using the Maximum Likelihood (ML) method with the General Time Reversible model (Nei and Kumar 2000) in Mega 12 software (Kumar et al. 2024). Bootstrap analysis with 1000 iterations was performed to assess phylogenetic robustness (Felsenstein 1985).

### 2.2 Ancestral State Reconstruction of Host Associations

To infer the evolutionary history of host associations within Spirurina, we performed ancestral state reconstruction (ASR) (Huelsenbeck et al. 2003) on the phylogenetic tree described above. Each species was assigned to one of nine ecological host‐use categories based on published information: Amphibia, Cockroach, Crustacean, Mammal, Millipede, Other host, Other insect, Reptile, or Free‐living (Tables S1 and S2). Actual host refers to the host species from which each nematode specimen was isolated, either in this study or in the original description. Entry host category represents the ecological category used for ASR and corresponding to the primary intermediate host in species with complex life cycles. For nematodes with direct life cycles, the host from which the specimen was isolated was treated as the definitive host, and its category was used directly. For species with complex life cycles, the assigned category reflects the primary intermediate host, as inferred from ecological traits or published reports. Remarks indicate whether each species has a direct or complex life cycle (Tables S1 and S2).

To obtain a time‐scaled phylogeny with contemporaneous tips, we transformed the rooted tree into an ultrametric chronogram using the penalized‐likelihood implemented in chronos from the *ape* package (*λ* = 1) (Paradis et al. 2004). This procedure ensured that all terminal taxa were aligned to a common temporal axis prior to ASR.

We reconstructed the evolution of host‐use categories using stochastic character mapping implemented in make.simmap (phytools) (Sanderson 2002; Revell 2012) under an equal‐rates (ER) transition model with 1000 simulations. To incorporate the biological hypothesis that the common ancestor of Spirurina was free‐living, we specified a fixed root prior (*π*) in which the free‐living state was assigned probability 1 and all other states probability 0. This forced the root of the tree to be reconstructed as free‐living in all simulations.

Posterior probabilities of host states at internal nodes were summarized using describe.simmap, and the most probable host category along each branch segment was visualized on the chronogram using plotSimmap. Node pies represent the posterior probability distribution of host states at each internal node.

### 2.3 Molecular Phylogenetic Analysis of the Infraorder Oxyuridomorpha

Among the species used in the molecular phylogenetic analysis of the suborder Spirurina, those belonging to the infraorder Oxyuridomorpha were selected for detailed analysis. Species with available sequence data for the D2/D3 28S rRNA and 18S rRNA genes were obtained from the NCBI database (Table S3).

In addition, we included sequences from 11 newly identified species of parasitic nematodes belonging to the infraorder Oxyuridomorpha, which used millepede, cockroach, and other insect as host with a direct life cycle. We identified the host arthropod species and isolated nematodes for morphological classification, gene sequencing, and infrapopulation surveys (data not shown). Genomic DNA extraction and two marker gene sequencing were conducted from three individuals per sequence as described above. Taxonomic identification at the family or genus level was determined based on D2/D3 28S rRNA sequence similarity, morphological characteristics, and host information of the closest matches, as described above. As sequences were 100% identical within each species, representative sequences have been deposited in the NCBI database and are summarized in Table S4, along with corresponding host information.

Seven species of Ascaridoidea (*Ascaris lumbricoides*, *Parascaris equorum*, *Toxascaris leonina*, *Anisakis simplex*, *Pseudoterranova decipiens*, *Krefftascaris sharpiloi*, and *Heterocheilus tunicatus*), seven species of Rhigonematoidea (*Trachyglossoides* sp.1, *Rhigonema thysanophora*, *R. disparovis*, *R. naylae*, *R. ingens* , *Xystrognathus phrissus*, and *Obainia* sp.), and six species of Ransomnematoidea (*Heth pivari*, *H. taybaci*, *Ransomnema bravoae*, *Brumptaemilius justini*, *Cattiena trachelomegali*, and *Insulanema longispiculum*) were used as outgroup taxa (aligned dataset is available as Supporting Information 2). The phylogenetic tree of the infraorder Oxyuridomorpha was generated using the ML method with the Jukes‐Cantor model (Jukes and Cantor 1969) using the Mega 11 software (Tamura et al. 2021). Bootstrap analysis with 1000 iterations was performed to assess the phylogenetic robustness (Felsenstein 1985).

## 3 Results

### 3.1 Molecular Phylogenetic Analysis of the Suborder Spirurina

**FIGURE 2:**
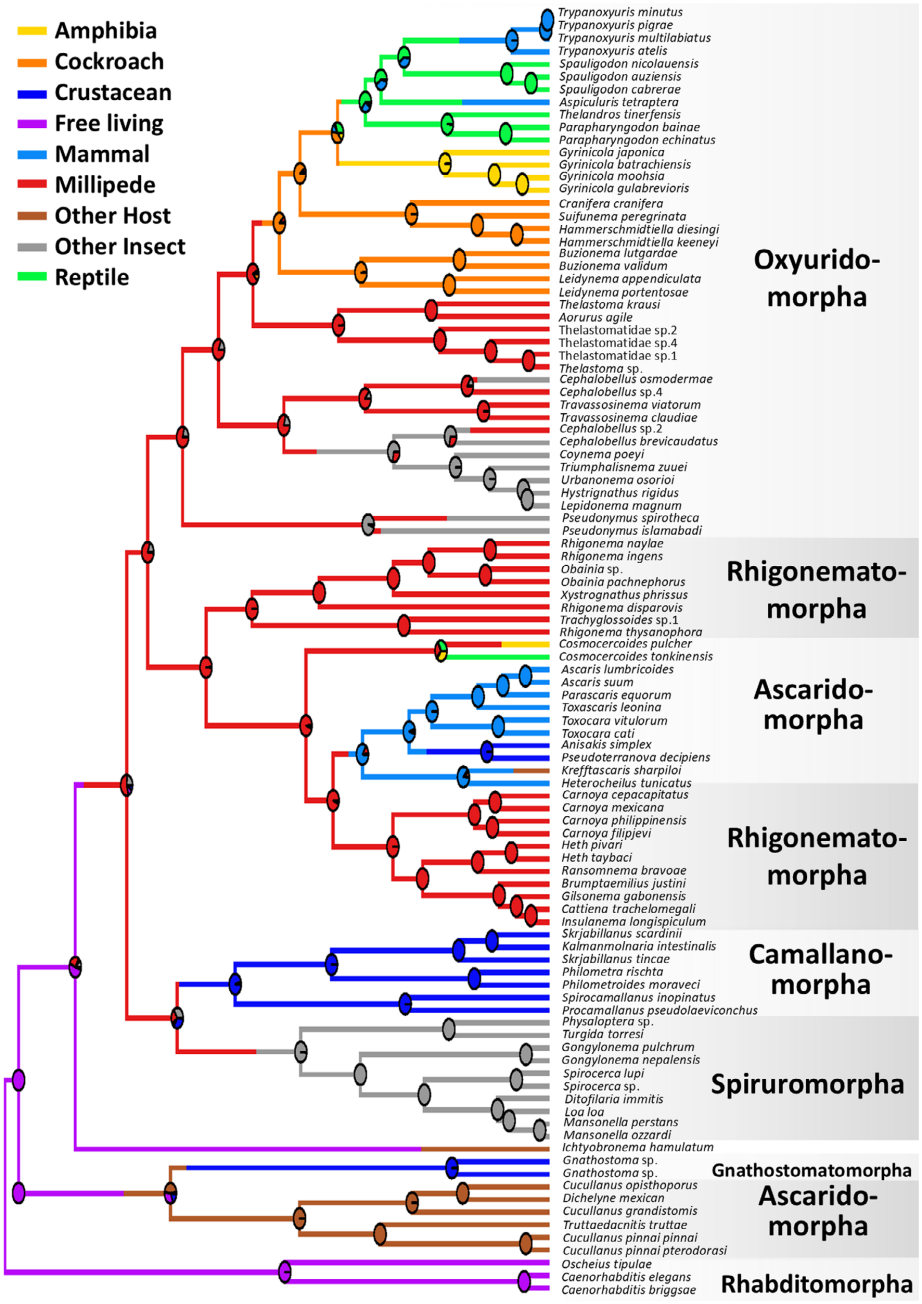
Phylogenetic tree of the suborder Spirurina with ancestral state reconstruction (ASR) of host‐use categories. The chronogram was inferred from a concatenated dataset of the 18S ribosomal RNA gene (649 bp) and the D2/D3 region of the 28S ribosomal RNA gene (544 bp). Branch colors represent the most probable host‐use category inferred by stochastic character mapping under an equal‐rates (ER) model. Node pies indicate the posterior probability distribution of host states at each internal node. Host‐use categories include Amphibia, Cockroach, Crustacean, Mammal, Millipede, Other host, Other insect, Reptile, and Free‐living. The root node was reconstructed as Free‐living with a posterior probability of 1.0. Major infraorders within Spirurina (Oxyuridomorpha, Rhigonematomorpha, Ascaridomorpha, Camallanomorpha, Spiruromorpha, and Gnathostomatomorpha) are indicated on the right.

The suborder Spirurina is traditionally classified into six infraorders: Oxyuridomorpha, Rhigonematomorpha, Ascaridomorpha, Gnathostomatomorpha, Spiruromorpha, and Camallanomorpha (Blaxter and Koutsovoulos 2015). Representative species from these infraorders were selected, and a phylogenetic tree was constructed using the D2/D3 28S rRNA gene and the 18S rRNA gene (Figure 2).

The ancestral state reconstruction (ASR) strongly supported a free‐living origin of Spirurina. The root node of the chronogram was reconstructed as Free‐living with a posterior probability of 1.0, consistent with the rooting strategy based on the three free‐living outgroup taxa ( *C. elegans* , *C. briggsae* , *O. tipulae*). From this free‐living ancestor, at least two independent origins of parasitism were inferred across the Spirurina phylogeny.

A major transition from free‐living to parasitic lifestyles occurred at the base of the large clade containing Oxyuridomorpha, Rhigonematomorpha, Ascaridomorpha, Camallanomorpha, and Spiruromorpha. Our ASR indicated that the earliest parasitic ancestor in this clade was most likely associated with millipedes, suggesting that millipedes served as an important initial host group for the emergence of parasitism in this lineage (Figure 2).

Within this clade, Spiruromorpha and Camallanomorpha appear to have maintained relatively stable host associations since their early divergence. Ascaridomorpha—including *Ascaris*, which directly infects terrestrial mammals, and *Anisakis*, which uses copepods and krill as first intermediate hosts and marine mammals as definitive hosts—was placed within Rhigonematomorpha (Rhigonematoidea and Ransomnematoidea), a group that primarily parasitizes millipedes. Our ASR further revealed that members of the superfamily Cosmocercoidea—traditionally classified as Ascaridomorpha (Alcantara et al. 2022; Chen, Zhang, Feng, et al. 2020; Chen, Zhang, and Li 2020) and parasitic in amphibians and reptiles—are nested within Rhigonematomorpha, suggesting that they originated from millipede‐parasitic ancestors. Oxyuridomorpha exhibits substantially diverse and consists of two major clades: Thelastomatoidea, which transitioned from millipede parasitism to parasitism of cockroaches and other arthropods, and Oxyuroidea, which independently evolved vertebrate parasitism, including in amphibians, reptiles, and mammals.

A second, independent origin of parasitism was inferred within Gnathostomatomorpha and a subset of nematodes previously classified within Ascaridomorpha (Seuratoidea). The ancestral state in this lineage involved invertebrate intermediate hosts, indicating a parasitic origin distinct from the millipede‐associated transition described above. Among Gnathostomatomorpha, only two *Gnathostoma* sp. have the D2/D3 28S rRNA and the 18S rRNA gene sequences available, and no other genera are represented. This highlights the need for additional sampling to clarify the evolutionary history of this clade. *Ichthyobronema hamulatum*, traditionally placed in Ascaridomorpha (Seuratoidea, Quimperiidae), showed an unstable placement between Spiruromorpha and Gnathostomatomorpha in our phylogenetic tree, further emphasizing the need for expanded taxon sampling.

### 3.2 Molecular Phylogenetic Analysis of the Infraorder Oxyuridomorpha

**FIGURE 3:**
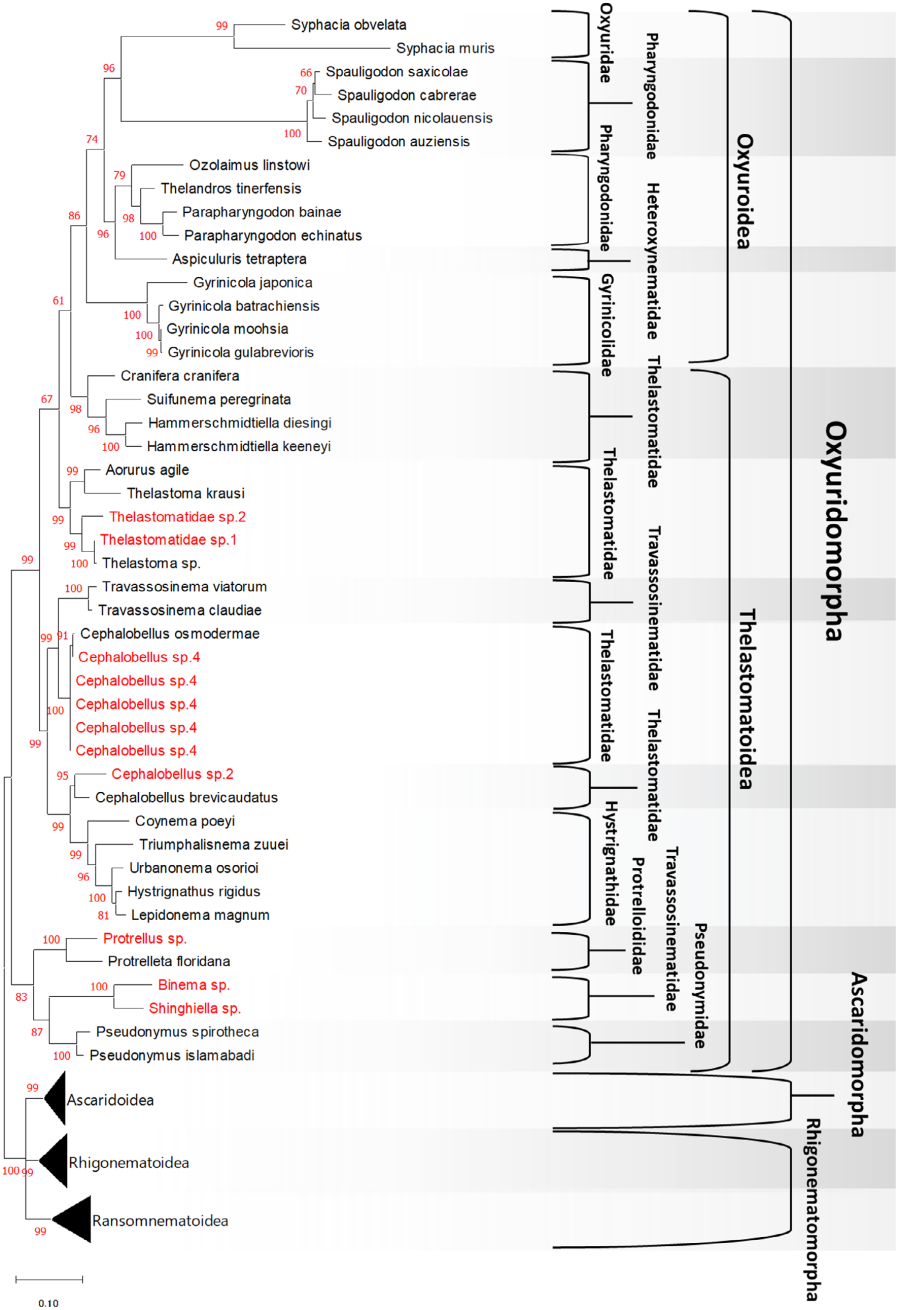
Phylogenetic tree of the infraorder Oxyuridomorpha, constructed using the maximum likelihood method based on a concatenated dataset of the 18S ribosomal RNA gene (727 bp) and the D2/D3 region of the 28S ribosomal RNA gene (571 bp). Bootstrap support value (%) from 1000 replicates are shown at branch points. New sequences obtained in this study (Table S4) are indicated in red. All families within Oxyuridomorpha are distinguished using different shades of gray.

Sequence data for 10 families, 37 species belonging to Oxyuridomorpha were obtained from the NCBI database (Table S3), and additional sequence data for three families and 11 species were newly obtained in this study (Table S4). Molecular phylogenetic analysis of the infraorder Oxyuridomorpha confirmed its division into two superfamilies: the vertebrate‐parasitic Oxyuroidea and the invertebrate‐parasitic Thelastomatoidea, consistent with previous reports (Adamson 1994; Nagae et al. 2021; Hodda 2022) (Figure 3).

The superfamily Thelastomatoidea is shown to be paraphyletic to the superfamily Oxyuroidea and is highly diverse, with parasitic nematodes utilizing various invertebrate hosts in a state of disorder, showing little evidence of co‐evolution with their hosts (See discussion, Figure 5). Based on the nematode species examined in this study, the family Thelastomatidae was divided into four phylogenetically distinct groups: (1) a clade comprising *Cranifera* spp., *Suifunema* spp., and *Hammerschmidtiella* spp.; (2) a clade containing *Aorurus* spp. and *Thelastoma‐related* taxa; (3) a clade consisting of *Cephalobellus* spp.; and (4) a separate clade formed by another group of *Cephalobellus* spp. (Figure 3). This phylogenetic tree also indicates that the vertebrate‐parasitic Oxyuroidea diverged from within Thelastomatidae. The family Travassosinematidae has now been largely divided into two distinct groups: one parasitizing millipedes, such as *T. claudiae* (Morffe and Hasegawa 2017) and *T. viatorum* (Morffe et al. 2023), which is closely related to the *Cephalobellus* spp. (Figure 3); and another parasitizing mole crickets, including the genera *Binema* spp. and *Singhiella* spp., which are closely related to the family Pseudonymidae and Protrelloididae (Figure 3).

Within the superfamily Oxyuroidea, the family Pharyngodonidae, which mainly parasitizes lizards, is paraphyletic to the mammal‑parasitic family Oxyuridae (Figure 3). *Aspiculuris tetraptera*, a mouse pinworm belonging to the family Heteroxynematidae, was also placed within Pharyngodonidae. Additionally, the parasitic nematode genus *Gyrinicola*, which infects amphibian tadpoles, represents the earliest lineage to diverge within the superfamily Oxyuroidea (Figure 3).

## 4 Discussion

### 4.1 Origin of the Suborder Spirulina

Whereas previous studies have primarily focused on resolving higher‐level relationships within Nematoda using ribosomal or phylogenomic datasets (Blaxter et al. 2000; Holterman et al. 2019; Qing et al. 2025), our study complements these efforts by explicitly integrating host‐association data and ancestral state reconstruction to trace the evolutionary pathways of parasitism within Spirurina.

**FIGURE 4:**
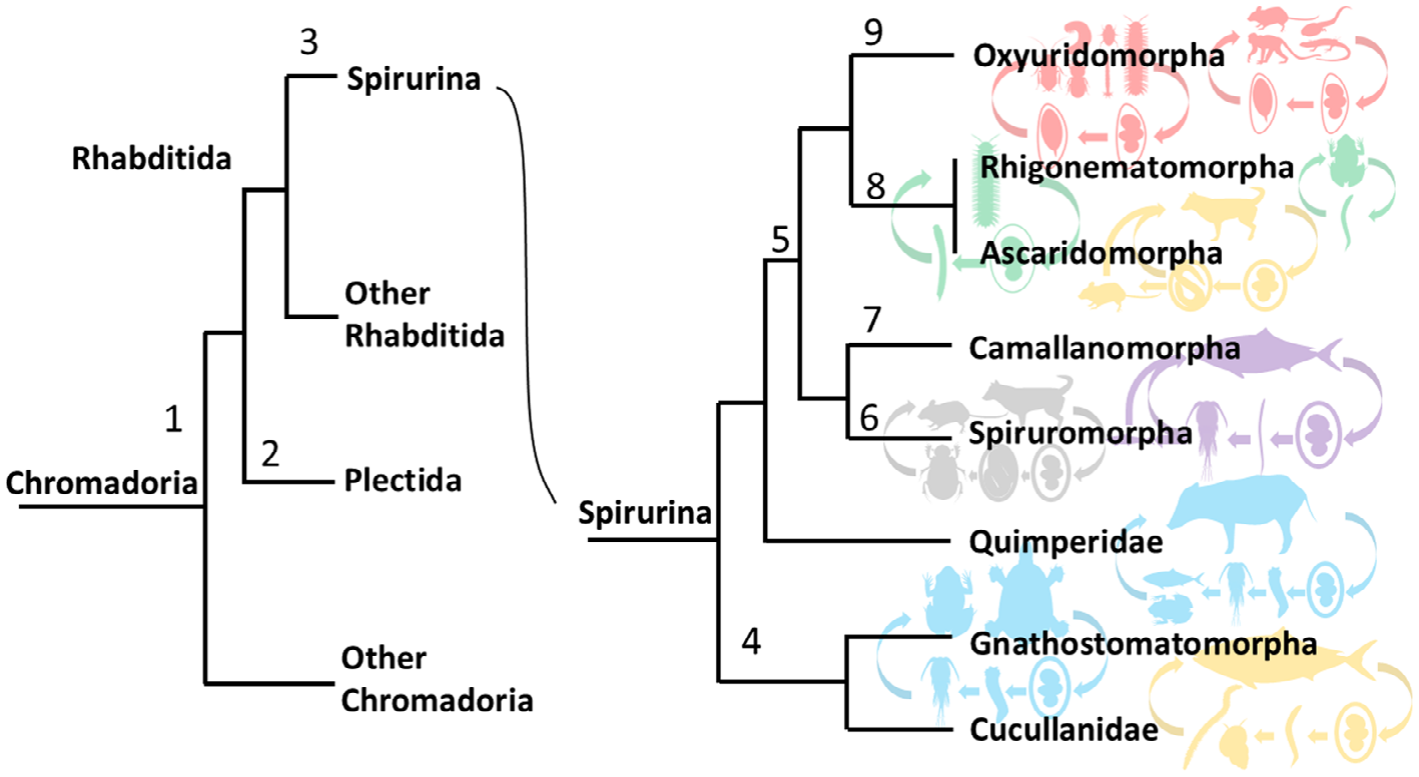
Phylogenetic tree of the subclass Chromadoria and the suborder Spirurina, created with reference to Figure 1 of Blaxter and Koutsovoulos (2015) and incorporating the phylogenetic results of this study (Figure 2). Each numbered point represents a putative evolutionary event during divergence: 1. Divergence of free‐living marine nematodes within the subclass Chromadoria. 2. Adaptation of certain lineages to terrestrial environments, giving rise to the ancestor of Plectida. 3. Separation of the ancestor of Spirurina from other Rhabditida. 4. Acquisition of parasitism in aquatic invertebrates, followed by the evolution of complex life cycles with fish as definitive hosts. 5. Acquisition of parasitism in millipedes. 6. Acquisition of parasitism in insects, followed by the evolution of complex life cycles with terrestrial vertebrates as definitive hosts. 7. Independent acquisition of parasitism in aquatic invertebrates, again followed by the evolution of complex life cycles within fish as definitive hosts. 8. Maintenance of millipede parasitism, with some lineages subsequently acquiring vertebrate parasitism and evolving complex life cycles. 9. Retention of a simple infection cycle while acquiring the ability to parasitize both invertebrates and vertebrates.

Nematodes are believed to have originated in the marine environment, and phylogenetic analysis suggests that terrestrial free‐living nematodes, such as those in the order Rhabditida, evolved from free‐living marine nematodes within the subclass Chromadoria (Blaxter and Koutsovoulos 2015) (Figure 4). Nematodes are thought to have adapted to terrestrial life by inhabiting interstitial water in soils, and by the early Devonian period, they are believed to have lived in soil alongside arthropod soil‐dwelling organisms (van Straalen 2021; Dunlop and Garwood 2018). According to molecular phylogenetic analysis, the ancestors of both terrestrial plants and arthropods are believed to have already colonized land during the Cambrian period (Fernández et al. 2018; Morris et al. 2018), and it is possible that nematodes in the order Rhabditida also colonized land during the same period. Early parasitic nematodes of the suborder Spirurina within the order Rhabditida are thought to have been bacterivorous, like their sister group of free‐living soil nematodes, and to have fed on the gut microbiota of their hosts (Adamson 1994).

### 4.2 Evolution of Parasitism in the Infraorder Gnathostomatomorpha and the Superfamily Seuratoidea

One of the earliest parasitic groups within Spirurina includes Gnathostomatomorpha and the superfamily Seuratoidea (currently classified within Ascaridomorpha), both of which possess complex life cycles requiring multiple hosts, with freshwater or marine invertebrates serving as the first intermediate hosts. In Gnathostomatomorpha, larvae of the superfamily Gnathostomatoidea hatch and are ingested by copepods as the first intermediate hosts, before infecting definitive hosts such as weasels or turtles via second intermediate or paratenic hosts, including fish, amphibians, and snakes (Ando et al. 1992; Hedrick 1935). In Seuratoidea, larvae or eggs are ingested by intermediate hosts such as polychaetes or gastropods, and hatched larvae may also utilize specific fish species as intermediate hosts before reaching the definitive host (Køie 2000, 2001; Choudhury and Cole 2019; Shears and Kennedy 2005). Although most nematodes belonging to Cucullanidae exhibit marine parasitic ecologies, *Truttaedacnitis truttae*, which parasitizes salmonid fish, is known to use freshwater snails as intermediate hosts (Choudhury and Cole 2019). This suggests that the lineage may have originated in freshwater environments and subsequently expanded into marine ecosystems.

*Ichthyobronema hamulatum*, currently classified within Ascaridomorpha (superfamily Seuratoidea, family Quimperiidae), presents an intriguing case. Although its life cycle has not been fully described, previous studies from Lake Baikal report that this species occurs predominantly in Scorpaeniformes fishes, and that benthic invertebrates such as dipteran larvae and mayflies likely function as intermediate hosts (Baldanova et al. 2021). In our phylogenetic analysis, however, *I. hamulatum* did not cluster with any other members of Ascaridomorpha or Seuratoidea, instead forming a solitary lineage without clear affinity to established infraorders.

This ambiguous placement may reflect the limited availability of molecular data for lineages closely related to *Ichthyobronema*, particularly within Gnathostomatomorpha and Seuratoidea, both of which represent early‐diverging parasitic groups within Spirurina. Without broader taxon sampling—including additional genera from Gnathostomatomorpha, Seuratoidea, and related aquatic parasitic lineages—it remains difficult to determine whether *I. hamulatum* represents an independent parasitic radiation or a deeply divergent member of an existing infraorder.

### 4.3 Evolution of Parasitism in the Infraorders Camallanomorpha and Spiruromorpha

In the infraorder Camallanomorpha, larvae released directly from viviparous females are ingested by copepods, which serve as intermediate hosts. The superfamily Camallanoidea and the family Philometridae use fish as definitive hosts, whereas the family Dracunculidae parasitizes mammals and reptiles (Stromberg and Crites 1974; Cleveland et al. 2018; Ogawa et al. 2023). In the infraorder Spiruromorpha, freshwater species infect fish or waterfowl after their eggs are ingested by intermediate hosts such as crustaceans, bivalves, or caddisfly larvae (Moravec and Scholz 1994; Moravec and Huffman 2001). Terrestrial species, by contrast, release eggs in feces, which are ingested by intermediate hosts before infecting mammals (Schell 1952; Petri 1950; Mowlavi et al. 2009; Kudo et al. 2003; Rojas et al. 2017).

Across these parasitic ecologies, aquatic nematodes consistently require intermediate hosts, and all four infraorders—Ascaridomorpha, Gnathostomatomorpha, Camallanomorpha, and Spiruromorpha—utilize crustaceans. Notably, copepods act as intermediate hosts for both Camallanomorpha and Gnathostomatomorpha, and as paratenic hosts for primitive reptile‐ and fish‐parasitic nematodes in the family Heterocheilidae (Ascaridomorpha). This pattern suggests that the dependence on copepods as an infection route is an ancestral feature widely shared among Spirurina lineages exhibiting complex life cycles that require multiple hosts.

Copepods and krill, dominant primary consumers in marine and freshwater ecosystems, form a major component of zooplankton biomass and play a crucial role in food webs (Abo‐Taleb et al. 2020; Reid and Williamson 2010; Tremblay et al. 2020). Nematodes using these organisms as intermediate or paratenic hosts establish infection when ingested as larvae, regardless of taxonomic group. Experimental studies confirm that copepods prey on free‐living nematodes (Muschiol et al. 2008). Thus, copepods and krill are both abundant prey for higher consumers and predators of smaller primary consumers, making them key transmission pathways for parasitic nematodes.

### 4.4 Evolution of Parasitism in the Infraorders Rhigonematomorpha and Ascaridomorpha

Among the three families within Rhigonematomorpha, Rhigonematidae, Carnoyidae and Hethidae, all groups use millipedes as definitive all groups use millipedes as definitive hosts. In the family Rhigonematidae, eggs excreted outside the host can hatch, and the larvae or eggs are believed to be transmitted orally to the host (Adamson and van Waerebeke 1985; Nagae et al. 2021). Our phylogenetic analysis further indicates that some lineage traditionally classified within Ascaridomorpha form an early‐diverging group together with Gnathostomatomorpha, whereas others, including *Ascaris*, *Anisakis*, and *Toxocara*, are nested within Rhigonematomorpha (Figure 2). In addition, the superfamily Cosmocercoidea, also traditionally placed within Ascaridomorpha, is recovered within Rhigonematomorpha in our tree (Figure 2). Cosmocercoidea has been classified as Ascaridomorpha since the time of morphological taxonomy, and recent molecular studies have compared *Ascaris* species with Cosmocercoidea (Alcantara et al. 2022; Chen, Zhang, Feng, et al. 2020; Chen, Zhang, and Li 2020). This study, together with the comprehensive Spirurina phylogeny of Nagae et al. (2021), clearly resolves the positions of Cosmocerca and Ascaridomorpha. Members of the family Cosmocercidae are commonly found in the intestines of adult frogs and toads and are known to spread via percutaneous infection (Chen, Zhang, Feng, et al. 2020; Chen, Zhang, and Li 2020; Chen et al. 2021; Liu et al. 2020; González et al. 2021; Alcantara et al. 2022; Ni et al. 2022). Their infection cycle is relatively simple: ovoviviparous larvae are excreted in the feces of the host frog and subsequently infect new frog hosts. This pattern suggests that Cosmocercidae may have undergone host switching from millipedes to amphibian, potentially mediated by predator–prey interactions (Nagae et al. 2021).

**FIGURE 5:**
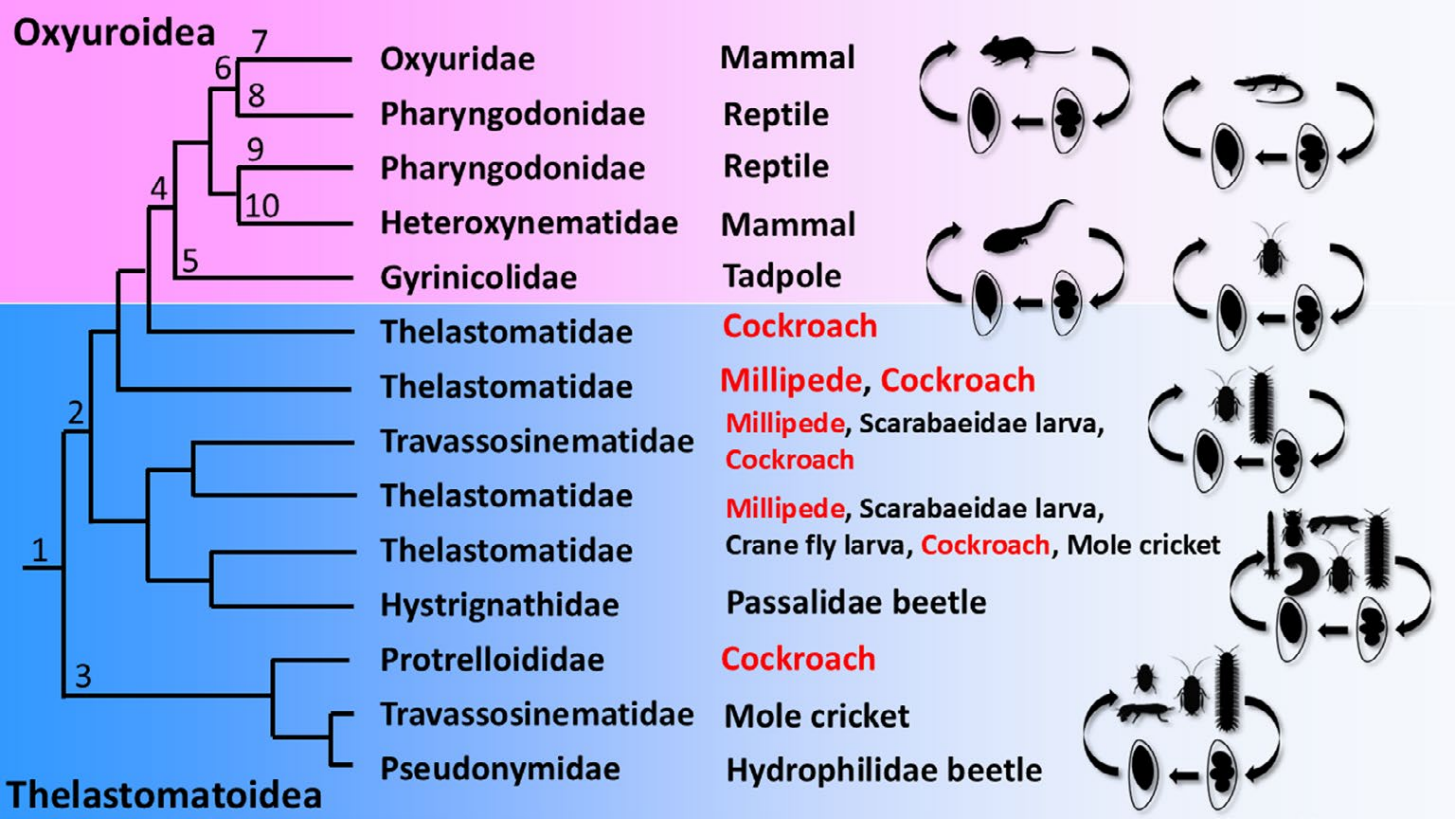
Phylogenetic tree of the suborder Oxyuridomorpha, created with reference to Figure 3 of this study. Each numbered point indicates a putative evolutionary event during divergence: 1. Maintain millipede parasitism through a simple infection cycle involving oral ingestion of eggs. 2. Diversification through host‐switching among arthropods. 3. Diversification through host‐switching among arthropods. 4. Acquisition of parasitism in vertebrates. 5. Acquisition of parasitism in tadpoles. 6. Development of host specificity. 7. Acquisition of parasitism in mammals. 8. Acquisition of parasitism in reptiles. 9. Acquisition of parasitism in reptiles. 10. Acquisition of parasitism in mammals.

*Dujardinascaris*, a genus of parasitic nematodes in the family Heterocheilidae, infects reptiles and manatees as definitive hosts. Their larvae hatch from eggs and are ingested by intermediate hosts such as fish, with copepods serving as paratenic hosts (Mahmoud 1999; Li et al. 2018). Marine parasitic nematodes within the suborder Ascaridomorpha, which infect a variety of marine vertebrates including bony fish, sharks, rays, whales, and pinnipeds, are also well documented (Roca‐Geronès et al. 2018; Li et al. 2018). In contrast, terrestrial members of Ascaridomorpha use rodents as paratenic hosts, and in some cases, larvae are transmitted from mother to offspring through breast milk. Some species also exhibit a more complex life cycle, in which larvae develop within the egg to the infective stage, are then orally ingested by mammalian hosts, migrate from the small intestine to the lungs for growth, and subsequently return to the small intestine to mature (Sprent 1956; Coati et al. 2004; Asaolu et al. 2002) (Figure 5).

### 4.5 Evolution of Parasitism in the Infraorder Oxyuridomorpha

The life cycle of nematodes in the suborder Oxyuridomorpha is generally similar across species. In cockroach‐parasitic nematodes, eggs excreted with feces are orally ingested through hosts' coprophagic behavior, whereas in mammals, pinworms lay eggs around the anus, which are then ingested orally through grooming and/or coprophagic behaviors (Hugot et al. 1999; Ozawa et al. 2016; Ozawa and Hasegawa 2018). Coprophagy is also common in arthropods, such as millipedes. Because nematodes in the superfamily Thelastomatoidea infect a wide range of decomposer hosts, including cockroaches, crickets, Passalidae beetles, Hydrophilidae beetles, Scarabaeidae beetle larvae, Lucanidae beetle larvae, and crane fly larvae, it is assumed that Thelastomatoidea species parasitizing hosts other than cockroaches also have similar life cycles (Adamson 1994; Culliney 2013).

In the infraorder Oxyuridomorpha, the superfamily Thelastomatoidea represents a more primitive group than the superfamily Oxyuroidea, with the latter having evolved from Thelastomatoidea nematodes (Figure 5). Thelastomatoidea species exhibit low host specificity and are believed to be capable of infecting a wide range of hosts (Jex, Hu, et al. 2006; Jex, Schneider, et al. 2006; Jex et al. 2007; Ozawa et al. 2016; Ozawa and Hasegawa 2018). This suggests that Thelastomatoidea, with its broad host range, served as a basal group that facilitated diversification through host‐switching. Examining the overall phylogenetic tree of Thelastomatoidea shows parasitism in millipedes and cockroaches occurs across multiple taxa (Figure 5). This indicates that the ancestor of Oxyuridomorpha initially adapted to parasitism in millipedes or cockroaches before expanding to a wide range of invertebrate hosts.

As it expanded its host range and diversified/speciated among various arthropods, some species might adapt to tadpoles and lizards as hosts. Tadpoles are omnivorous, consuming both plant and animal matter, including algae and detritus, which makes them markedly different from adult amphibians, which are predominantly carnivorous (Montaña et al. 2019; Halliday 2008). Although lizards are primarily carnivorous, many species also display omnivorous tendencies by consuming plant matter (Cooper and Vitt 2002). Based on this, tadpoles can be considered more similar in diet to cockroaches than to other amphibians or lizards.

The fossil record of Anura dates back to the Early Jurassic (201–174 million years ago), while tadpoles specifically appear in the Middle Jurassic (168–161 million years ago). Molecular clock estimates suggest that Anura may have diverged from other groups as early as the Carboniferous period (approximately 310 million years ago) (Chuliver et al. 2024; Lee and Anderson 2006). Because the ancestors of cockroaches have remained morphologically unchanged since the Carboniferous (Grimaldi and Engel 2005), it is possible that the ancestors of Oxyuroidea nematodes originated during this period by switching hosts from ancestral cockroaches to tadpoles (Adamson 1994; Ozawa and Hasegawa 2018).

The family Pharyngodonidae is a paraphyletic group within the family Oxyuridae, with one lineage clustering with Heteroxynrmatidae and the other with Oxyuridae (Figure 5). Comparing these two separated Pharyngodonidae lineages, the former group, which parasitizes various reptiles such as lizards and snakes, shows low geographical polymorphism, whereas the latter group, which parasitizes only specific reptiles, exhibits high geographical polymorphism, indicating differences in host specificity (Falk and Perkins 2013). Reports have shown that the population size of Oxyuridae nematodes in mammalian hosts is regulated by the host's acquired immune system (Michels et al. 2006). Nematodes of the infraorder Oxyuridomorpha feed on gut microbiota distributed throughout the intestine, and in humans, this microbiota is also regulated by the immune system (Magalhaes et al. 2007). Within the family Oxyuridae, co‐evolution with hosts from the orders Rodentia and Primates has been documented (Robles and Navone 2010; Hugot et al. 1999). From this, it can be inferred that Oxyuridae nematodes inhabiting the gut microbiota of mammals are subject to immune regulation, and their ability to resist such regulation enables their parasitism.

In the family Pharyngodonidae, which may have originated from the cockroach‐parasitic nematode group, causes of maternal transmission have been reported, in which host larvae lick the eggshell immediately after hatching (Goldberg and Bursey 1992; Malysheva 2016; Troyer 1982). Lizards have also been observed grooming their bodies, including their cloacal region, and licking their feces, much like mammals (Prieto and Ryan 1978). Thus, similar to Thelastomatoidea, Pharyngodonidae nematodes establish their life cycle through coprophagic behavior, and lizard‐parasitic species are believed to retain many characteristics of their closest ancestors, the cockroach‐parasitic nematodes. Therefore, the host specificity observed in Oxyuridae is thought to have been acquired by Pharyngodonidae after their evolutionary shift to lizard parasitism.

## 5 Conclusion

In this study, we reconstructed the evolutionary history of parasitism within Spirurina by integrating molecular phylogenetics with ecological information. Our ASR indicates that the earliest parasitic ancestor expanded its host associations from invertebrates to vertebrates over evolutionary time, and that parasitic strategies evolved from simple, single‐host infection cycles to complex life cycles requiring intermediate hosts. Several taxa were recovered as paraphyletic or polyphyletic, but their placements correspond closely with parasitic ecology, suggesting that taxonomic revision will be possible with broader sampling. Millipedes appear to have played a pivotal role in the early diversification of terrestrial parasitism, whereas copepods represent a key ancestral intermediate host group underpinning the evolution of complex, multi‐host life cycles in aquatic lineages. Together, these findings provide a concise evolutionary framework for understanding the diversification of parasitic strategies across Spirurina.

## Author Contributions

**Seiya Nagae:** conceptualization (equal), data curation (supporting), formal analysis (equal), funding acquisition (equal), investigation (equal), methodology (equal), resources (supporting), software (equal), validation (equal), visualization (equal), writing – original draft (equal), writing – review and editing (supporting). **Koichi Hasegawa:** conceptualization (equal), data curation (lead), formal analysis (equal), funding acquisition (equal), investigation (equal), methodology (equal), project administration (lead), resources (lead), software (equal), supervision (lead), validation (equal), visualization (equal), writing – original draft (equal), writing – review and editing (lead).

## Funding

This work was supported by Japan Science and Technology Agency (JPMJSP2158), Chubu University.

## Conflicts of Interest

The authors declare no conflicts of interest.

## Supporting information


**Supporting Information 1:** Sequence alignment for Figure 2. This file contains the aligned nucleotide sequences of all nematode species used for the phylogenetic analysis of the suborder Spirurina shown in Figure 2.

**Supporting Information 2:** Sequence alignment for Figure 3. This file contains the aligned nucleotide sequences of all nematode species used for the phylogenetic analysis of the infraorder Oxyuridomorpha shown in Figure 3.

**Table S1:** List of the sequences used in the phylogenetic analysis of the suborder Spirurina from the NCBI database.
**Table S2:** List of the new sequences used in the phylogenetic analysis of the suborder Spirurina.
**Table S3:** List of the sequences used in the phylogenetic analysis of the infraorder Oxyuridomorpha from NCBI database.
**Table S4:** List of the new sequences used in the phylogenetic analysis of the infraorder Oxyuridomorpha.

## Data Availability

All data generated or analyzed during this study are included in this published article or are available from the corresponding author on reasonable request. DNA sequences of nematode isolates are available in the GenBank repository under the following accession numbers: PV642592‐642596, PV642599‐642605, PV642607‐642616. Sequence alignments used in Figures 2 and 3 are available as Supporting Information Data 1 and 2.
